# Allelic Dropout Is a Common Phenomenon That Reduces the Diagnostic Yield of PCR-Based Sequencing of Targeted Gene Panels

**DOI:** 10.3389/fgene.2021.620337

**Published:** 2021-02-01

**Authors:** Anna G. Shestak, Anna A. Bukaeva, Siamak Saber, Elena V. Zaklyazminskaya

**Affiliations:** ^1^Medical Genetics Laboratory, Petrovsky National Research Center of Surgery, Moscow, Russia; ^2^Cardiac Electrophysiology Research Center, Rajaie Cardiovascular Medical and Research Center, Iran University of Medical Sciences, Tehran, Iran

**Keywords:** allelic dropout, cardiomyopathy, next-generation sequencing, Sanger sequencing, diagnostic yield, DNA diagnostics

## Abstract

Primary cardiomyopathies (CMPs) are monogenic but multi-allelic disorders with dozens of genes involved in pathogenesis. The implementation of next-generation sequencing (NGS) approaches has resulted in more time- and cost-efficient DNA diagnostics of cardiomyopathies. However, the diagnostic yield of genetic testing for each subtype of CMP fails to exceed 60%. The aim of this study was to demonstrate that allelic dropout (ADO) is a common phenomenon that reduces the diagnostic yield in primary cardiomyopathy genetic testing based on targeted gene panels assayed on the Ion Torrent platform. We performed mutational screening with three custom targeted gene panels based on sets of oligoprimers designed automatically using AmpliSeq Designer® containing 1049 primer pairs for 37 genes with a total length of 153 kb. DNA samples from 232 patients were screened with at least one of these targeted gene panels. We detected six ADO events in both IonTorrent PGM (three cases) and capillary Sanger sequencing (three cases) data, identifying ADO-causing variants in all cases. All ADO events occurred due to common or rare single nucleotide variants (SNVs) in the oligoprimer binding sites and were detected because of the presence of “marker” SNVs in the target DNA fragment. We ultimately identified that PCR-based NGS involves a risk of ADO that necessitates the use of Sanger sequencing to validate NGS results. We assume that oligoprimer design without ADO data affects the amplification efficiency of up to 0.77% of amplicons.

## Introduction

In recent years, the study of the genetic causes of monogenic diseases has evolved from a basic science research area into widely accepted clinical testing protocols with substantial impacts on diagnostics and clinical decision-making (Ackerman et al., 2011). Primary cardiomyopathies (CMP) are monogenic but multi-allelic disorders with dozens of genes involved in pathogenesis (Hershberger et al., 2018). The prevalence of clinically expressed and hypertrophic cardiomyopathy (HCM) gene carriers has been greatly underestimated and could be as high as 1:200 (Semsarian et al., 2015).

The implementation of NGS approaches has resulted in more time- and cost-efficient DNA diagnostics of cardiomyopathies. However, the diagnostic yield of genetic testing for each subtype of CMP fails to exceed 60% (Hershberger et al., 2018). Negative results obtained by genetic testing do not rule out the presence of genetic disease because our knowledge about the molecular pathogenesis of disease is still evolving. Moreover, the technical limitations of all known techniques of DNA/RNA analysis and variant interpretation contribute to incomplete results. Alternative sequencing approaches such as capillary Sanger sequencing confirm the genetic variants found by NGS methods to increase the reliability of the DNA test results (Baudhuin et al., 2015).

Allelic dropout (ADO) is a common phenomenon that reduces the efficiency of PCR-based targeted sequencing. It was first described in 1991 as a “partial amplification failure,” causing a potential source of misdiagnosis for both dominant and recessive diseases (Navidi and Arnheim, 1991). The practical importance of the ADO phenomenon was originally shown in 1997 by Lissens and Sermon in a case of preimplantation genetic diagnosis of cystic fibrosis wherein the heterozygous ΔF508 mutation in the CFTR gene was not detected in 25% of mutant blastomeres (Lissens and Sermon, 1997). The ADO phenomenon involves selective allele amplification during the polymerase chain reaction (PCR) thermocycling process. The presence of single nucleotide variants (SNVs) in the forward and/or reverse oligoprimer binding sites may lead to the complete or partial lack of amplification of the single allele, while the second one may “drop” out during the PCR process. In such cases, SNVs causing ADO are usually located closer to the 3′ end of the oligoprimer binding site (Martins et al., 2011).

Bi-directional capillary Sanger sequencing and high-throughput semiconductor sequencing approaches are routinely used for cross-validation of genetic findings (Baudhuin et al., 2015; Di Resta and Ferrari, 2018). Both approaches are PCR-based, share similar limitations, and may be negatively impacted by ADO. However, the incidence of ADO events in these PCR-based diagnostic assays remains unknown.

The aim of this study was to demonstrate that ADO is a common phenomenon influencing the diagnostic yield of targeted gene panel testing of primary CMPs on the Ion Torrent platform with follow-up verification by Sanger sequencing.

## Materials and Methods

We performed genetic testing on DNA samples from 232 patients diagnosed with inherited cardiomyopathies in clinical centres. This study was performed in accordance with the 1964 Helsinki declaration, its later amendments and local ethics committee. Written informed consent was obtained from all individual participants included in the study. DNA samples were extracted from venous blood using Quick-DNA Miniprep Plus Kit (Zymo Research Corp., Irvine, CA, USA) according to the manufacturer's instructions.

Mutational screening was performed using three custom targeted gene panels with two sets of oligoprimers designed automatically using Ion AmpliSeq Designer® (Thermo Fisher Scientific, Waltham, MA, USA) containing 1,049 primer pairs for 37 genes, with a total length of 153 kb. More detailed characteristics of each target genes panels are presented in Supplementary Table 1. Manufacturer grouped primers for each panel in 2 pools. Libraries preparation was performed using Ion AmpliSeq™ Library Kit 2.0 according to the manufacturer's instructions (Thermo Fisher Scientific). Sequencing was performed on Ion 314™ and Ion 316™ chips using high-throughput semiconductor sequencing on an Ion PGM™ System according to the manufacturer's instructions (Thermo Fisher Scientific). Average reads per amplicon were 192, mean coverage with at least 20 reads−94.7%, mean coverage with at least 100 reads−79.51%. Data from the Ion PGM™ System were processed with CoverageAnalysis and VariantCaller plugins available within licensed Torrent Suite Software 5.6.0 and Ion Reporter Software (Thermo Fisher Scientific). NGS sequencing reads were visualized using the Integrative Genomic Viewer (IGV) tool (Robinson et al., 2011) using hg19 as a reference genome. All DNA samples were screened with at least one of the targeted gene panels mentioned.

Rare genetic variants detected by NGS were verified via bi-directional capillary Sanger sequencing on an ABI 3730XL DNA Analyzer according to the manufacturer's instructions (Thermo Fisher Scientific). Alternative pairs of oligoprimers flanking the coding and adjacent intronic regions of the 37 genes were designed for PCR using open-source PerlPrimer (Marshall, 2004). The PCR protocol and annealing temperature of the primers were determined experimentally. The results of direct Sanger sequencing were visualized using Chromas 2 software (Technelysium Pty Ltd, South Brisbane, Australia).

All archival direct Sanger sequencing chromatograms were involved in the study to track the ADO phenomenon. Genetic variants found by NGS were visually compared with Sanger sequencing chromatograms, noting the possible loss of heterozygosity or underrepresentation of alternative alleles. To reveal the cause of ADO, forward- and reverse-primer binding sites were analysed using the Genome Aggregation Database (gnomAD) (Karczewski et al., 2020). In order to exclude only one allele amplification, all amplicons with noted or suspected ADO cases were re-sequenced with alternative non-overlapping oligoprimer pairs.

All genetic variants newly detected in this study were registered in public database ClinVar (https://www.ncbi.nlm.nih.gov/clinvar/). List of variants with accession numbers is summarized in Supplementary Table 2.

## Results

We performed mutational screening on 232 DNA samples from patients diagnosed with different types of inherited CMPs. The DNA samples were screened with at least one of the three targeted gene panels.

We found that three ADO cases occurred during sequencing on the IonTorrent platform and three occurred during capillary Sanger sequencing. In the targeted gene panels, ADO led to underrepresentation/loss of marker variants in NGS reads (Table 1). The Sanger sequencing chromatograms revealed a dropout of the allele due to the loss of heterozygosity of the already detected (“marker”) SNV (Table 1). Control capillary re-sequencing using additional alternative oligoprimers confirmed the true allelic status.

**Table 1:** Revealed allelic dropout events in sequencing data.

**Gene**	**Amplicon position (hg19)**	**SNV causing ADO**	MAF^*^	**Marker variant(s)**	**Zygosity**	**Type of event**	**Sequencing platform with ADO occured**	**Re-sequencing platform**	**Number of confirmed cases**
*SCN5A*	chr3:38597041-38597372	c.4542+89C>T	0.087	c.4516C>T (p.P1506S)	Hetero	Missing wt (c.4516C>T hemizygous)	Sanger	Ion Torrent	1
*PKP2*	chr12:32948847-32949434	c.2300-195A>G	0.139	c.2489+13_2489+14insC, p.2489+72_73delinsA^*^, c.2489+109A>G	Hetero	Missing wt (^*^detected as homozygous)	Sanger	Ion Torrent	9
*DSP*	chr6:7572015-7572438	c.1904-49T>A	0.411	c.2091A>G (p.G697G)	Hetero	Missing	Sanger	Ion Torrent	1
*LDB3*	chr10:88466446-88466568	p.T351A (c.1051A>G)	0.0006	p.T351A (c.1051A>G)	Hetero	Underrepresented (3%)	Ion torrent	Ion Torrent, Sanger	1
*LDB3*	chr10:88451719-88451942	p.K251R (c.752A>G)	0.0014	p.K251R (c.752A>G)	Hetero	Underrepresented (11%)	Ion torrent	Sanger	1
*SCN1B*	chr19:35524839-35525003	p.R214Q (c.641G>A)	0.0042	c.641G>A (p.R214Q)^*^, c.744C>A (p.S248R), c.749G>C (p.R250T)	Hetero	Missing, (^*^under-represented, 5%)	Ion torrent	Sanger	2

* *MAF in European (non-Finnish) population based on gnomAD data*.

We identified the cause of ADO in all six cases (Table 1). In the targeted genes, ADO was caused by rare or unique genetic variants in the oligoprimer binding sites; in capillary Sanger sequencing, all ADO cases occurred due to common SNVs.

**Figure 1:**
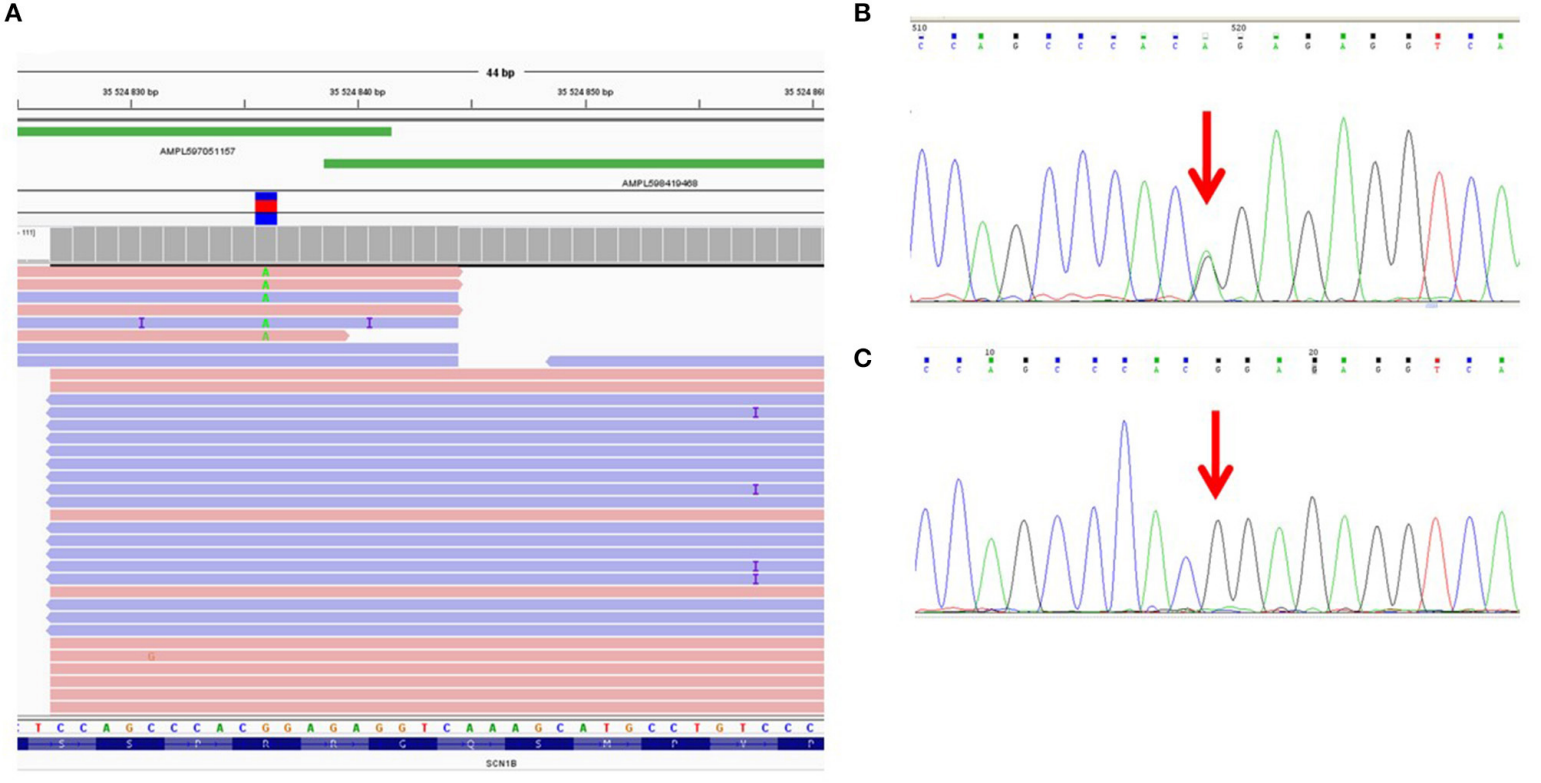
Allelic dropout in the *SCN1B* gene in DNA sample of proband NRF124. **(A)** Ion Torrent PGM sequencing results. Genetic variant c.641G>A is detected on only one of two overlapping amplicons. **(B)** Fragment of the Sanger sequencing chromatogram showing the presence of heterozygous variant c.641G>A in the proband (arrow). **(C)** Fragment of the Sanger sequencing chromatogram obtained with oligoprimers identical to AmpliSeq.

We found that the ADO phenomenon may lead not only to the complete loss of an allele but also to the underrepresentation of the “marker” variant in NGS reads. For example, the heterozygous rare missense variant c.641G>A (p.R214Q) in the *SCN1B* gene was detected in only 1 of 2 overlapping amplicons by target IonTorrent sequencing and was represented in 5% of all reads (Figure 1A). This caused the loss of two missense variants, —c.744C>A (p.S248R) and c.749G>C (p.R250T)—and only reads with the wild-type allele were displayed. The presence of all three linked heterozygous missense variants—c.641G>A (p.R214Q), c.744C>A (p.S248R), and c.749G>C (p.R250T)—in the *SCN1B* gene in the DNA sample was confirmed by control Sanger sequencing (Figure 1B).

To reproduce this case of ADO in a single (i.e., non-multiplexed) PCR, we performed a single control PCR with two oligoprimers designed by AmpliSeq and flanking genomic region chr19:35524839-35525003 (the corresponding target region of the *SCN1B* gene). This amplicon was sequenced separately by capillary Sanger sequencing and the loss of the allele containing the c.641G>A (p.R214Q) variant was reproduced (Figure 1C).

**Figure 2:**
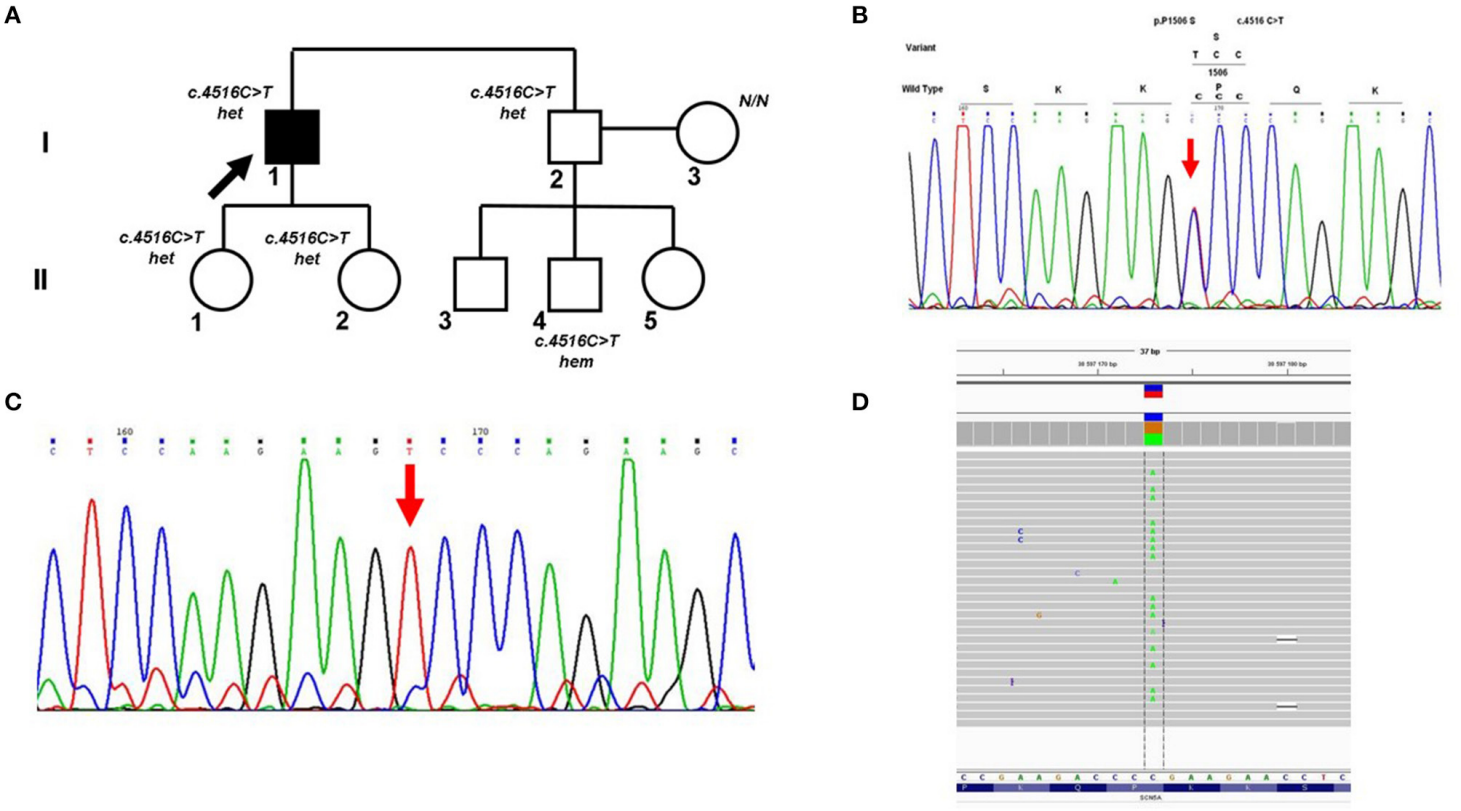
Allelic dropout in the *SCN5A* gene in family with Brugada syndrome. **(A)** Pedigree of the family; Arrow marks the proband. Het, heterozygous; hem, hemizygous. **(B)** Fragment of the Sanger sequencing chromatogram showing the presence of heterozygous variant c.4516C>T in the proband (arrow). **(C)** Fragment of the Sanger sequencing chromatogram showing the presence of hemizygous variant c.4516C>T in the II-4 family member (arrow). **(D)** Control re-sequencing on the IonTorrent platform showing the family member II-4 is a carrier of heterozygous variant c.4516C>T.

Cross-validation of DNA diagnostic results using an alternative sequencing approach (capillary Sanger sequencing was performed first as a basic method) allowed us to identify a case of allelic dropout in the *SCN5A* gene in an Iranian family with Brugada syndrome (Figure 2A). Heterozygous missense variant c.4516C>T (p.P1506S) in exon 26 of the *SCN5A* gene was found by capillary Sanger sequencing in DNA samples of the proband (Figure 2B) and proband's brother. However, further cascade familial screening revealed this missense variant in the proband's nephew in the hemizygous state (Figure 2C). Theoretically, the hemizygous state of this c.4516C>T variant in the II-4 family member may involve alternative explanations such as consanguinity in the family or *de novo* deletion of the *SCN5A* gene in the maternal allele, but it did not fit the clinical phenotype and family history. Dropout of the wild-type allele was the most reliable explanation.

Manual analysis of oligoprimer binding sites using gnomAD revealed the presence of a common genetic variant c.4542+89C>T [total minor allele frequency (MAF) 0.098] in the 3′-end of the R-primer which caused ADO. This heterozygous SNV was detected in DNA samples of patient II-4 and his mother by PCR-RFLP analysis using an additional pair of oligoprimers flanking this region. Absence of genetic variant c.4516C>T (p.P1506S) in the mother's DNA sample was confirmed by capillary Sanger sequencing with two independent oligoprimers and PCR-RFLP analysis. Control re-sequencing on the IonTorrent platform showed that family member II-4 is a carrier of heterozygous c.4516C>T (p.P1506S) (Figure 2D).

In one case, we detected ADO when comparing results from two consecutive targeted gene panel sequencing assays with overlapping gene spectra and different oligoprimers encompassing the target regions of the *LDB3* gene. A variant of unknown significance—c.1051A>G (p.T351A)—in the *LDB3* gene in sample ARVD19 was detected only by panel I (“Genes encoding desmosomal and associated proteins”) (Supplementary Figure 1A) but not in panel II (“Genes encoding sarcomeric and associated proteins”) (Supplementary Figure 1B). This SNV was also confirmed by control capillary sequencing (Supplementary Figure 1C).

We also found that ADO may occur not only due to localization of SNVs in the 3′-end of oligoprimer binding sites, but also close to the 5′-end. Deep intronic variant c.2300-195A>G in the *PKP2* gene was located close to the 5′-end and led to ADO in nine samples studied (Table 1).

We found that ADO is a non-consistent process even in the same DNA sample. For example, three consecutive PCR capillary sequencing runs with sample ARVD16 yielded one positive result (heterozygous variant c.2091A>G was detected) and two negative results (complete loss of the c.2091A>G variant). This ADO event was caused by the intronic variant c.1904-49T>A located at the 3′-end of the primer-binding site.

All amplicons with identified ADO events were carefully re-sequenced using alternative oligoprimer pairs and selective allele amplification was confirmed in all cases (Table 1).

## Discussion

Currently, ADO is a known limitation of PCR-based molecular diagnostic approaches. Caused by different mechanisms, the single allele amplifies exclusively or pre-dominantly, leading to overrepresentation of homozygosity (Wang et al., 2012).

Following the automatization of all molecular diagnostic procedures—from primer design to variant detection, calling, and interpretation—that has increased the amount of samples tested simultaneously, the awareness of ADO events should also be increased. The Clinical Laboratory Standards Institute (CLSI) Guidelines recommend that assay development and quality control should include measures aimed at both detecting allelic dropout and minimizing its occurrence (CLSI, 2012).

In this study, six ADO events were identified in both PCR-based sequencing platforms. The causes of these events were also revealed in all cases. On the IonTorrent platform, ADO events were caused by rare or unique genetic variants in the oligoprimer binding sites. On the capillary sequencing platform, ADO events were caused by common SNVs in the oligoprimer binding sites (Table 1). As a result of such selective amplification, we observed partial hemizygosity or underrepresentation of the heterozygous genetic variants in the NGS results. Some of these underrepresented variants may be filtered automatically during NGS data processing. Cross-validation of the genetic findings revealed by one sequencing platform with an alternative approach is a powerful method to decrease the rate of false-positive results in genetic testing. However, there is no universally-accepted method to decrease—let alone effectively detect—partial hemizygosity due to allelic dropout. It seems that resequencing of the region of interest by two independent oligoprimer pairs remains the “gold standard” of DNA diagnostics.

There are 2 types of causes of the ADO phenomenon described in literature (Wang et al., 2012):

(1) “Sample-specific” causes due to the quality of the DNA sample or the low DNA concentration. Such ADO cases are found in forensic diagnostics, where only fragmented or degraded DNA is available, as well as in preimplantation diagnostics, where genotyping is performed on DNA extracted from one blastomere;
(2) “Locus-specific” causes due to the characteristics of the locus under investigation. The presence of single nucleotide polymorphisms (SNPs) in oligoprimer binding sites of forward and/or reverse primers disturbs the specificity of the complementary interaction between the oligonucleotide and the target DNA sequences, leading to the lack of oligoprimer hybridization, and elongation of the amplicon.

All ADO events revealed in this study involved locus-specific causes due to the characteristics of individual loci in normal concentrations of DNA.

In cases of locus-specific allelic dropout, the causal SNV in the oligoprimer binding site is usually located close to the 3′-end of the oligoprimer. This was initially reported in a study by Martins et al. (2011). The authors used the rs2247836 variant (MAF = 0.403 in the European population and 0.323 in the African population) in the intron 4 of the *PAH* gene to evaluate the probability of ADO depending on SNV location in oligoprimer binding sites. Four alternative variants of forward oligoprimers were designed containing the SNV in the 3rd, 5th, and 7th positions of the 3′-end of the oligoprimer. Sanger sequencing was performed for patients carrying the heterozygous genetic variant rs2247836 and mutation p.Arg158Gln in exon 5 of the *PAH* gene. Loss of heterozygosity was detected for all positions of the ADO-causing SNV. The authors recommended careful consideration during primer design of the rare/common SNVs in areas within 7 nucleotides of the 3′-end of oligoprimers. We found that the presence of SNVs close to the 5′-end of oligoprimers may also cause ADO events. Convincing data suggest that any polymorphic position within the oligoprimer sequence potentially reduces the accuracy of DNA diagnostics (Martins et al., 2011).

Data from 30,769 reported genotypes for eight mutations involved in four diseases show that, on average, allele dropout/drop-in potentially leading to misdiagnosis occurred in 0.44% of genotype results (Blais et al., 2015).

We re-analysed the oligoprimer binding sites containing SNVs within overlapping amplicons and found additional amplicons that may be missing due to ADO events. The presence of SNVs in these fragments was exhibited in the NGS reads as underrepresented variants and/or was revealed in isolated reads (Table 2).

**Table 2:** SNVs localized in the oligoprimers' binding sites and threatening PCR efficiency.

**Gene**	**Amplicon position (hg19)**	**SNV potentially affecting PCR efficiency**	AF^*^ (gnomAD)
*DSP*	chr6:7574805-7575146	c.2298-85C>T	0.605
*DSP*	chr6:7579369-7580822	c.3085-115C>T	0.676
*SCN5A*	chr3:38622472-38622620	c.3183A>G (p.E1061E)	0.890
*LDB3*	chr10:88466471-88466552	c.1074C>T (p.A358A)	0.044
*LDB3*	chr10:88493015-88493177	c.^*^450G>A	0.001556
*FLNC*	chr7:128480667-128480889	c.1614C>T (p.Y538Y)	0.039
*FLNC*	chr7:128487631-128487861	c.4404T>C (p.D1468D)	0.999

* *Allele Frequency in European (non-Finnish) population based on gnomAD data*.

We hypothesize that the risk of ADO would increase with the number of target genes and overall panel size because it would increase number of oligoprimer pairs to cover. Potential causes of ADO (SNVs) were found in 4 of 521 amplicons in panel 1 (0.77%). There are increasing numbers of studies that discuss the importance of ADO in DNA diagnostic procedures (Tester et al., 2006; Coulet et al., 2010; Medlock et al., 2012; Rossetti et al., 2012; Lam and Mak, 2013; Shmukler et al., 2013; Rhees et al., 2014; Blais et al., 2015; Proost, 2016). This phenomenon was detected in oncogenetics (*BRCA1/2* testing), inborn metabolic disease genotyping (*FAH* testing), hematology research etc (Lam and Mak, 2013; Shmukler et al., 2013; Jeong et al., 2019). We surmise that the actual number of ADO events remains unknown and may significantly exceed the events actually detected. The risk of the negative impacts of possible polymorphic sites in automatically generated primer sequences on sequencing results remains high. It depends on the number of overlapping amplicons. It seems that underrepresentation of genetic variants in NGS reads is not dependent on the read depth. Jeong et al. demonstrated a reproducible ADO phenomenon during 3 consecutive re-sequencing by Ion S5 with read depth from 1985 to 8608 (Jeong et al., 2019).

Allelic dropout can lead to underdetection of the rare variants within missing amplicons and increase false negative results rate in diagnostic setting, and can cause mistaken assignment of heterozygous genotypes as homozygotes with underestimation of the observed heterozygosity in population studies. The simple strategy to restore allelic drop out could be a repeated genotyping with non-overlapping pairs of oligoprimers. But in daily practice replicate genotyping is costly. Increasing the tiling density of amplicons would be also helpful but it requires more oligos for design and synthesis, and also noticeably increases the assay price. Another way could be to improve automatic primer design tools with continuously updating dbSNP and gnomAD data to omit inclusion of the SNPs into the primer sequences. Analysis the secondary structure of primer and template sequences would also be important for the design algorithm (Lam and Mak, 2013).

Regular updates on SNV distribution and prevalence in the human genome and improvement in primer design algorithms would greatly improve the diagnostic yield of molecular genetic testing.

In conclusion, PCR-based sequencing technologies such as next-generation sequencing and Sanger sequencing are widely used in clinical practice. Despite their high throughput and constantly improving efficiency, limitations remain for the application of these technologies.

All PCR-based methods involve the risk that ADO will decrease the diagnostic yield of genetic testing because of undetectable, potentially pathogenic variants. We demonstrate that ADO is a common phenomenon in both NGS and Sanger sequencing results.

Theoretically, ADO may affect up to 0.77% of amplicons. It seems that the actual rate of ADO may be even higher and is dependent on the number of oligoprimer pairs. Specific software that incorporates updates on the distribution of SNVs to avoid ADO resulting from automatic oligoprimer design would substantially increase the accuracy of molecular research.

Oligoprimer sequences are available upon request.

## Data Availability Statement

The datasets for this article are not publicly available due to concerns regarding participant/patient anonymity. Requests to access the datasets should be directed to the corresponding author. The ClinVar reference numbers for the variants discussed in the article are present in the article text and/or Supplementary Material.

## Ethics Statement

The studies involving human participants were reviewed and approved by the local Ethics Committee of Petrovsky National Research Center of Surgery (Moscow, Russia). The patients/participants provided their written informed consent to participate in this study.

## Author Contributions

AS, AB, and SS performed wet genetic investigation. AS performed data analysis and drafting the manuscript. EZ–management of the project, editing, and final approval of the manuscript. All authors read, discussed, and approved the manuscript as submitted.

## Conflict of Interest

The authors declare that the research was conducted in the absence of any commercial or financial relationships that could be construed as a potential conflict of interest.

## References

[B1] Ackerman M. J. Priori S. G. Willems S. Berul C. Brugada R. Calkins H.. (2011). HRS/EHRA expert consensus statement on the state of genetic testing for the channelopathies and cardiomyopathies: this document was developed as a partnership between the heart rhythm society (HRS) and the European heart rhythm association (EHRA). Europace 13, 1077–1109. 10.1093/europace/eur245 21810866

[B2] Baudhuin L. M. Lagerstedt S. A. Klee E. W. Fadra N. Oglesbee D. Ferber M. J. (2015). Confirming variants in next-generation sequencing panel testing by Sanger sequencing. J. Mol. Diagn. 17, 456–461. 10.1016/j.jmoldx.2015.03.004 25960255

[B3] Blais J. Lavoie S. B. Giroux S. Bussières J. Lindsay C. Dionne J.. (2015). Risk of misdiagnosis due to allele dropout and false-positive PCR artifacts in molecular diagnostics: analysis of 30,769 genotypes. J. Mol. Diagn. 17, 505–514. 10.1016/j.jmoldx.2015.04.004 26146130

[B4] CLSI (2012). Quality Management for Molecular Genetic Testing; Approved Guideline. CLSI document MM20-A. Wayne, PA: Clinical and Laboratory Standards Institute.

[B5] Coulet F. Pires F. Rouleau E. Lefol C. Martin S. Colas C.. (2010). A one-step prescreening for point mutations and large rearrangement in BRCA1 and BRCA2 genes using quantitative polymerase chain reaction and high-resolution melting curve analysis. Genet. Test. Mol. Biomarkers 14, 677–690. 10.1089/gtmb.2009.0183 20858050

[B6] Di Resta C. Ferrari M. (2018). Next generation sequencing: from research area to clinical practice. Ejifcc 29, 215–220. 30479607 PMC6247137

[B7] Hershberger R. E. Givertz M. M. Ho C. Y. Judge D. P. Kantor P. F. McBride K. L. (2018). Genetic evaluation of cardiomyopathy: a clinical practice resource of the American college of medical genetics and genomics (ACMG). Genet. Med. 20, 899–909. 10.1038/s41436-018-0039-z 29904160

[B8] Jeong T. D. Cho S. Y. Kim M. W. Huh J. (2019). Significant allelic dropout phenomenon of oncomine BRCA research assay on ion torrent S5. Clin. Chem. Lab. Med. 57, e124–e127. 10.1515/cclm-2018-0674 30367782

[B9] Karczewski K. J. Francioli L. C. Tiao G. Cummings B. B. Alföldi J. Wang Q.. (2020). The mutational constraint spectrum quantified from variation in 141,456 humans. Nature 581, 434–443. 10.1038/s41586-020-2308-7 32461654 PMC7334197

[B10] Lam C. W. Mak C. M. (2013). Allele dropout caused by a non-primer-site SNV affecting PCR amplification—A call for next-generation primer design algorithm. Clin. Chim. Acta 421, 208–212. 10.1016/j.cca.2013.03.014 23523590

[B11] Lissens W. Sermon K. (1997). Preimplantation genetic diagnosis: current status and new developments. Hum. Reprod 12, 1756–1761. 10.1093/humrep/12.8.1756 9308807

[B12] Marshall O. J. (2004). PerlPrimer: cross-platform, graphical primer design for standard, bisulphite and real-time PCR. Bioinformatics 20, 2471–2472. 10.1093/bioinformatics/bth254 15073005

[B13] Martins E. M. Vilarinho L. Esteves S. Lopes-Marques M. Amorim A. Azevedo L. (2011). Consequences of primer binding-sites polymorphisms on genotyping practice. Open J. Genet. 1, 15–17. 10.4236/ojgen.2011.12004

[B14] Medlock M. M. Tester D. J. Will M. L. Bos J. M. Ackerman M. J. (2012). Repeat long QT syndrome genetic testing of phenotype-positive cases: prevalence and etiology of detection misses. Heart Rhythm 9, 1977–1982. 10.1016/j.hrthm.2012.08.010 22885918

[B15] Navidi W. Arnheim N. (1991). Using PCR in preimplantation genetic disease diagnosis. Hum. Reprod. 6 836–849. 10.1093/oxfordjournals.humrep.a137438 1757524

[B16] Proost D. (2016). Improved molecular diagnostic testing for sudden cardiac death (Doctoral dissertation), Universiteit Antwerpen, Antwerp.

[B17] Rhees J. Arnold M. Boland C. R. (2014). Inversion of exons 1–7 of the MSH2 gene is a frequent cause of unexplained lynch syndrome in one local population. Fam. Cancer 13, 219–225. 10.1007/s10689-013-9688-x 24114314 PMC3984383

[B18] Robinson J. T. Thorvaldsdóttir H. Winckler W. Guttman M. Lander E. S. Getz G.. (2011). Integrative genomics viewer. Nat. Biotechnol. 29, 24–26. 10.1038/nbt.1754 21221095 PMC3346182

[B19] Rossetti S. Hopp K. Sikkink R. A. Sundsbak J. L. Lee Y. K. Kubly V.. (2012). Identification of gene mutations in autosomal dominant polycystic kidney disease through targeted resequencing. J. Am. Soc. Nephrol. 23, 915–933. 10.1681/ASN.2011101032 22383692 PMC3338301

[B20] Semsarian C. Ingles J. Maron M. S. Maron B. J. (2015). New perspectives on the prevalence of hypertrophic cardiomyopathy. J. Am. Coll. Cardiol. 65, 1249–1254. 10.1016/j.jacc.2015.01.019 25814232

[B21] Shmukler B. E. Mukodzi S. Andres O. Eber S. Alper S. L. (2013). Autosomal dominant overhydrated stomatocytosis associated with the heterozygous RhAG mutation F65S: a case of missed heterozygosity due to allelic dropout. Br. J. Haematol. 161, 602–604. 10.1111/bjh.12261 23406318

[B22] Tester D. J. Cronk L. B. Carr J. L. Schulz V. Salisbury B. A. Judson R. S.. (2006). Allelic dropout in long QT syndrome genetic testing: a possible mechanism underlying false-negative results. Heart Rhythm 3, 815–821. 10.1016/j.hrthm.2006.03.016 16818214

[B23] Wang C. Schroeder K. B. Rosenberg N. A. (2012). A maximum-likelihood method to correct for allelic dropout in microsatellite data with no replicate genotypes. Genetics 192, 651–669. 10.1534/genetics.112.139519 22851645 PMC3660999

