# Supplementary material for: Allelic Dropout Is a Common Phenomenon That Reduces the Diagnostic Yield of PCR-Based Sequencing of Targeted Gene Panels
Source: Front Genet. 2021 Feb 1;12:620337. doi: 10.3389/fgene.2021.620337 (PMC7901947; doi:10.3389/fgene.2021.620337)
Supplement: Supplementary file 1 [file Table_1.docx]

Supplementary Table 1. Characteristics of target genes panels used for mutational screening

| **Target genes panel** | **Number of genes** | **List of genes** | **Number of oligoprimers pairs** | **Mean amplicon length, bp** | **Mean overlap between consecutive amplicons, bp** | **Total targets size, bp** | **Number DNA samples tested** |
| --- | --- | --- | --- | --- | --- | --- | --- |
| Panel I: Genes encoding desmosomal and associated proteins | 16 | *PKP2, DSG2, DSP, DSC2, JUP, TMEM43, TGFB3, LMNA, DES, EMD, CRYAB, SCN5A, FLNC, PLN, CTNNA3, LDB3* | 521 | 240 | 30 | 94100 | 119 |
| Panel II:  Genes encoding sarcomeric and associated proteins | 10 | *MYBPC3, MYH7, TNNT2, TNNI3, TPM1, MYL2, MYL3, ACTC1, TAZ, LDB3* | 294 | 198 | 48 | 29000 | 10 |
| Panel III:  Genes encoding K^+^/Na^+^ ion channels | 11 | *SCN5A, SCN1B, SCN3B, SCN4B, SNTA1, KCNQ1, KCNH2, KCNJ2, KCNE1, KCNE2, KCNE3* | 234 | 195 | 33 | 30000 | 114 |
