# Supplementary material for: Allelic Dropout Is a Common Phenomenon That Reduces the Diagnostic Yield of PCR-Based Sequencing of Targeted Gene Panels
Source: Front Genet. 2021 Feb 1;12:620337. doi: 10.3389/fgene.2021.620337 (PMC7901947; doi:10.3389/fgene.2021.620337)
Supplement: Supplementary file 2 [file Table_2.docx]

Supplementary Table 2. List of genetic variants reported in this study

| **Gene** | **Genetic variant** | **Number rs** | **Accession number** **in ClinVar** |
| --- | --- | --- | --- |
| *SCN1B* | c.641G>A (p.R214Q) | rs66876876 | VCV000190847.10 |
| *SCN1B* | c.744C>A (p.S248R) | rs67701503 | VCV000138999.3 |
| *SCN1B* | c.749G>C (p.R250T) | rs67486287 | VCV000036756.3 |
| *SCN5A* | c.3183A>G (р.E1061E) | [rs7430407](https://www.ncbi.nlm.nih.gov/snp/rs7430407) | VCV000048297.9 |
| *SCN5A* | c.4516C>T (p.P1506S) | n/a | VCV000982413 |
| *SCN5A* | c.4542+89C>T | rs41315501 | n/a |
| *PKP2* | c.2300-195A>G | rs7138758 | VCV000672403.1 |
| *PKP2* | c.2489+13_2489+14insC | rs149968852 | VCV000045070.2 |
| *PKP2* | с.2489+72_73delinsA | rs61927769, rs774849612 | VCV000674370.1,  VCV000674371.1 |
| *PKP2* | c.2489+109A>G | rs12228193 | n/a |
| *DSP* | c.1904-49T>A | rs2076303 | VCV000259382.1 |
| *DSP* | c.2091A>G (p.G697G) | rs2076304 | VCV000044870.2 |
| *DSP* | c.2298-85C>T | [rs2259208](https://www.ncbi.nlm.nih.gov/snp/rs2259208) | VCV000672134.1 |
| *DSP* | c.3085-115C>T | [rs2744379](https://www.ncbi.nlm.nih.gov/snp/rs2744379) | VCV000672150.1 |
| *LDB3* | p.K251R (c.752A>G) | rs34423165 | VCV000036449.6 |
| *LDB3* | p.T351A (c.1051A>G) | [rs138251566](https://www.ncbi.nlm.nih.gov/snp/rs138251566) | VCV000036942.13 |
| *LDB3* | c.1074C>T (p.A358A) | rs45459491 | VCV000036443.3 |
| *LDB3* | c.*450G>A | rs45541534 | n/a |
| *FLNC* | c.1614C>T (p.Y538Y) | rs76046880 | VCV000129082.2 |
| *FLNC* | c.4404T>C (p.D1468D) | [rs2249128](https://www.ncbi.nlm.nih.gov/snp/rs2249128) | VCV000226641.1 |
