# Supplementary figures and images for: Allelic Dropout Is a Common Phenomenon That Reduces the Diagnostic Yield of PCR-Based Sequencing of Targeted Gene Panels
Source: Front Genet. 2021 Feb 1;12:620337. doi: 10.3389/fgene.2021.620337 (PMC7901947; doi:10.3389/fgene.2021.620337)

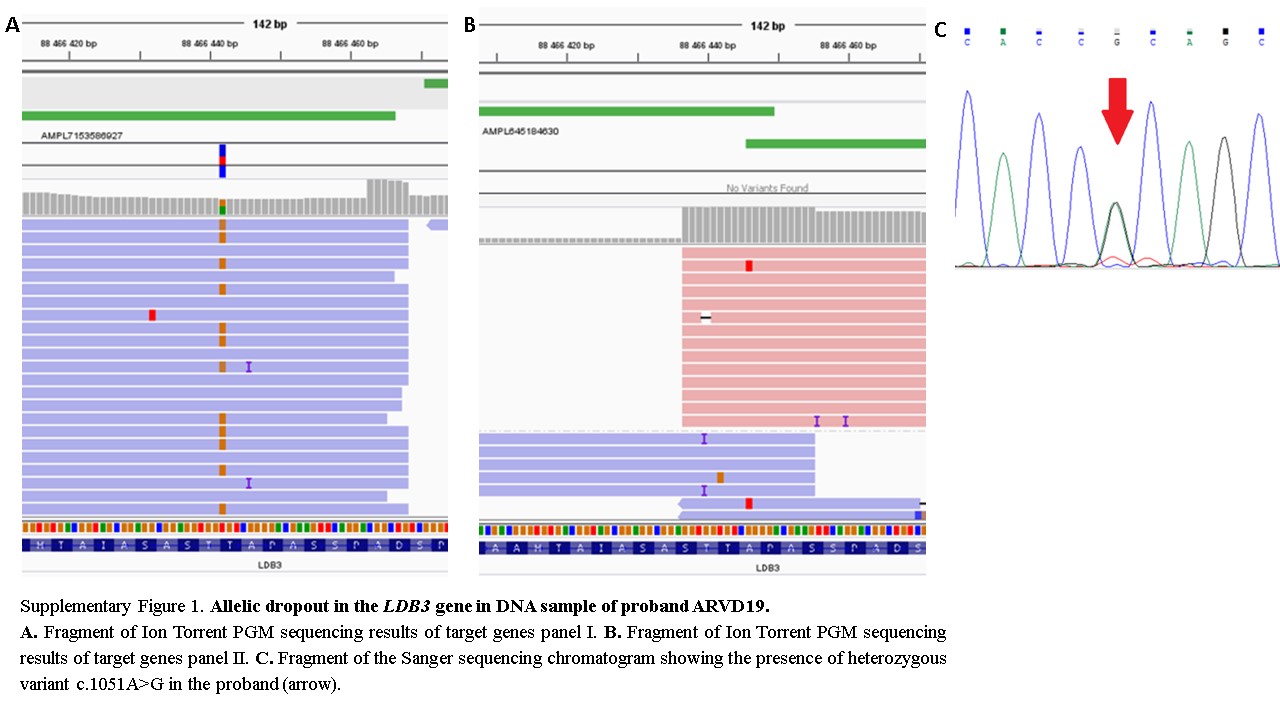

Supplement: Supplementary file 3 [file Image_1.JPEG]
